# A Multispecies Cluster of GES-5 Carbapenemase–Producing Enterobacterales Linked by a Geographically Disseminated Plasmid

**DOI:** 10.1093/cid/ciz1130

**Published:** 2019-11-20

**Authors:** Matthew J Ellington, Frances Davies, Elita Jauneikaite, Katie L Hopkins, Jane F Turton, George Adams, Jiri Pavlu, Andrew J Innes, Christopher Eades, Eimear T Brannigan, Jacqueline Findlay, Leila White, Frances Bolt, Tokozani Kadhani, Yimmy Chow, Bharat Patel, Siddharth Mookerjee, Jonathan A Otter, Shiranee Sriskandan, Neil Woodford, Alison Holmes

**Affiliations:** 1 National Institute for Health Research Health Protection Research Unit in Healthcare Associated Infections and Antimicrobial Resistance, Department of Medicine, Imperial College London, Hammersmith Hospital, London, United Kingdom; 2 Antimicrobial Resistance and Healthcare Associated Infections Reference Unit, National Infections Service, Public Health England, London, United Kingdom; 3 Imperial College Healthcare National Health Service Trust, London, United Kingdom; 4 Department of Infectious Disease Epidemiology, School of Public Health, Imperial College London, London, United Kingdom; 5 Microbiology, Royal Preston Hospital, Lancashire Teaching Hospitals National Health Service Foundation Trust, Preston, United Kingdom; 6 North West London Health Protection Team, Public Health England, London, United Kingdom; 7 Public Health Laboratory London, National Infections Service, Public Health England, London, United Kingdom

**Keywords:** GES-5 plasmid, Enterobacterales, outbreak, *Klebsiella oxytoca*

## Abstract

**Background:**

Early and accurate treatment of infections due to carbapenem-resistant organisms is facilitated by rapid diagnostics, but rare resistance mechanisms can compromise detection. One year after a Guiana Extended-Spectrum (GES)-5 carbapenemase–positive *Klebsiella oxytoca* infection was identified by whole-genome sequencing (WGS; later found to be part of a cluster of 3 cases), a cluster of 11 patients with GES-5–positive *K. oxytoca* was identified over 18 weeks in the same hospital.

**Methods:**

Bacteria were identified by matrix-assisted laser desorption/ionization–time of flight mass spectrometry, antimicrobial susceptibility testing followed European Committee on Antimicrobial Susceptibility Testing guidelines. Ertapenem-resistant isolates were referred to Public Health England for characterization using polymerase chain reaction (PCR) detection of GES, pulsed-field gel electrophoresis (PFGE), and WGS for the second cluster.

**Results:**

The identification of the first GES-5 *K. oxytoca* isolate was delayed, being identified by WGS. Implementation of a GES-gene PCR informed the occurrence of the second cluster in real time. In contrast to PFGE, WGS phylogenetic analysis refuted an epidemiological link between the 2 clusters; it also suggested a cascade of patient-to-patient transmission in the later cluster. A novel GES-5–encoding plasmid was present in *K. oxytoca*, *Escherichia coli*, and *Enterobacter cloacae* isolates from unlinked patients within the same hospital group and in human and wastewater isolates from 3 hospitals elsewhere in the United Kingdom.

**Conclusions:**

Genomic sequencing revolutionized the epidemiological understanding of the clusters; it also underlined the risk of covert plasmid propagation in healthcare settings and revealed the national distribution of the resistance-encoding plasmid. Sequencing results also informed and led to the ongoing use of enhanced diagnostic tests for detecting carbapenemases locally and nationally.

## Teaser

Whole-genome sequencing informed the detection of active and previously missed hospital clusters of *Klebsiella oxytoca* with GES-5; modes of spread were reinterpreted and GES-5 plasmid was found to be widely disseminated in other species of bacteria at remote locations.

Carbapenemase-producing Enterobacterales (CPE) have emerged globally over the last 2 decades [1]. They are endemic in some parts of the world including regions of Southern Asia [1, 2] and highly prevalent in parts of Southern Europe, Israel, and the United States [3–5]. In the United Kingdom (UK), selected regions report that up to 6% of inpatients are colonized [6–9], but overall prevalence of CPE in invasive infections remains low [10, 11].

Where infection occurs, multidrug resistance associated with CPE renders treatment difficult, patient morbidity is greater, and cases are more costly for healthcare facilities [3, 12, 13]. Immunocompromised patients have high mortality rates where appropriate treatment is delayed [14]. This may be improved by prompt diagnostics, but the diversity of carbapenemases in CPE makes testing challenging. Most assays seek only the 4 or 5 most widespread carbapenemase families: KPC, NDM, OXA-48–like, VIM, and IMP.

GES β-lactamases confer β-lactam resistance, but not all variants are carbapenemases [15]. GES carbapenemase variants have been reported in South America, the Middle East, Southeast Asia, Africa, and Europe [16]. The genes are horizontally transmissible between genera and species and variously found in *Acinetobacter baumannii* [17], *Pseudomonas aeruginosa* [18], and Enterobacterales [19]. Isolates have been identified during environmental sampling of hospital sinks and wastewater sites [19–21], thought to provide environmental reservoirs for the gene. Outbreaks due to GES-5 carbapenemase producers remain rare, although clonal outbreaks of *P. aeruginosa* [18] and *Enterobacter cloacae* [22] have been reported.

Detection of GES carbapenemases pose a challenge for laboratory medicine, raising the risk that cases are underascertained. Among Enterobacterales, GES carbapenemases alone can confer an ESBL-like phenotype with only low-level resistance to ertapenem and meropenem, which can be mistakenly attributed to impermeability, although GES-positive isolates with higher minimum inhibitory concentrations (MICs) have been noted [19]. Most current commercial molecular diagnostics do not detect GES genes.

This investigation describes an outbreak of GES-5–positive *Klebsiella oxytoca* in a hematology unit and the identification of a missed cluster in the same hospital a year earlier, and reveals the nationwide circulation of a GES-5 carbapenemase-encoding plasmid vector.

## MATERIALS AND METHODS

### Clinical Setting

The study center was a National Health Service (NHS) Healthcare Trust located in the west of London, which has approximately 1500 patient beds across 3 major hospitals, with approximately 1 125 000 patient contacts each year. At the time of the study, 2014–2016, the hematology unit was comprised of 3 wards, a day-care unit, and an outpatient department.

### Microbiological and Clinical Epidemiological Investigation

Bacterial isolates from clinical specimens and rectal swabs were identified by matrix-assisted laser desorption/ionization–time of flight mass spectrometry (Bruker Daltonics). Antimicrobial susceptibility testing was performed in accordance with European Committee on Antimicrobial Susceptibility Testing guidelines [23]. Selected isolates identified as ertapenem resistant by disk testing were additionally screened locally for carbapenemase production via Hodge test and polymerase chain reaction (PCR) for OXA-48, KPC, NDM, VIM, and IMP carbapenemase genes (Cepheid Xpert Carba-R). Ertapenem-resistant isolates of *K. oxytoca* were referred to Public Health England’s Antimicrobial Resistance and Healthcare Associated Infections (AMRHAI) Reference Laboratory for extended MIC determination via agar dilution and further characterization.

During this investigation (2015), improved phenotypic and/or rapid molecular detections for diverse (and phenotypically weak) carbapenemases were still in development. Carbapenemase activity was not initially identified (interpreted as an extended-spectrum β-lactamase [ESBL] hyperproducer) in the first GES-5 carbapenemase–producing *K. oxytoca* from this center (designated Kox-Z). However, the GES-5 gene was identified via whole-genome sequencing (WGS) and reported retrospectively as a GES-5 producer.

The referral of a further isolate 1 year later (Kox-A) also with unexplained ertapenem resistance triggered the prospective use of rapid PCR screening for the presence of GES alleles [24] in Enterobacterales from this center. The detection of carbapenemase activity was sought (including Kox-Z and Kox-A) via RAPIDEC CarbaNP (bioMérieux, France).

The epidemiological investigation included contact tracing in the hematology unit; all contacts were screened for rectal CPE carriage (Supplementary Methods). Potential retrospective cases from the preceding 18 months were identified by searching the microbiology data repository for ertapenem-intermediate or -resistant *K. oxytoca* isolates.

### Infection Control Measures

Detection of GES-5 in Kox-A (from patient A) prompted closure of the hematology ward to new patients and enhanced infection control procedures including contact precautions, enhanced chlorine cleaning, hand hygiene retraining for staff, use of personal protective equipment, equipment cleaning, and remedial works and shower repairs in selected bathrooms. Prospective admission and weekly screening for CPE was implemented for all patients in hematology. Admission and weekly screening in other high-risk areas (such as the intensive care unit) and risk factor-based screening of patients was already in place across the rest of the NHS Trust [25, 26].

### Molecular Characterization and Genomic Analysis

*Klebsiella oxytoca* isolates sent to the reference laboratory underwent *Xba*I pulsed-field gel electrophoresis (PFGE) analysis [27]. For WGS, extracted DNA from all isolates was sequenced on an Illumina HiSeq instrument (Supplementary Materials). Reads were submitted to the European Nucleotide Archive (number PRJEB30858; Supplementary Table 1).

Genomic analyses included multilocus sequence typing, detection of antimicrobial resistance genes and plasmid replicon types, and analysis of phylogenetic relatedness between isolates. To confirm the structure of the genetic vector encoding the GES-5 carbapenemase, long-read (Pacific Biosciences) sequencing of *E. cloacae* (from patient W) was used, and transfer of the GES-5–encoding plasmid from the initial *K. oxytoca* detected (from patient Z) to *E. coli* DH5α was followed by Illumina sequencing (see Supplementary Materials). Plasmids were compared with the best matches from the National Center for Biotechnology Information nucleotide database (see Supplementary Methods).

Isolates referred from other centers and NHS Trusts to AMRHAI for investigation of carbapenem resistance and found, via WGS, to harbor GES-5 and an IncQ plasmid replicon type (as were the outbreak isolates) were included as contextual isolates (Table 1) and for comparison of the GES-5 carbapenemase vector.

**Table 1.** Patient Characteristics, Sites, or Specimens That GES-5 Producers Were Isolated From and the Month of Isolation (Relative to the Study Hospital Investigation)

Patient/Sample Identifier	Clinical Area/Geographic Region	Isolate Species and Identifier	MLST	Specimen Site	Month of Isolation^a^	Temporal- spatial Clustering
A	Hematology	Kox-A Kox-A.2 Kox-A.3 Kox-A.4	ST138 ST138 ST138 ST138	Urine BAL Rectal screen blood	18–20	2 2 2 2
B	Hematology	Kox-B Kox-B.2	ST138 ST138	Rectal screen Rectal screen	19	2 2
C	Hematology	Kox-C	ST138	Rectal screen	19	2
D	Hematology	Kox-D	ST138	Rectal screen	19	2
E	Hematology	Kox-E	ST138	Rectal screen	19	2
F	Hematology	Kox-F	ST138	Rectal screen	19	2
G	Hematology	Kox-G	ST138	Rectal screen	20	2
H	Hematology	Kox-H	ST138	Rectal screen	20	2
I	Hematology	Kox-I Kox-I.2	ST138 ST138	Rectal screen Rectal screen	21	2 2
J	Hematology	Kox-J	ST138	Rectal screen	21	2
K	Hematology	Kox-K	ST138	Rectal screen	22	2
Z	Cardiothoracic/ITU	Kox-Z	ST138	Urine	6	1
Y	Cardiothoracic/ITU	Kox-Y	ST138	Wound	10	1
X	Cardiothoracic/ITU	Kox-X	ST138	Throat swab	10	1
W	Neurology	Ecl-W	ST66	Rectal screen	21	2a
V	Renal	Eco-V	ST101	Rectal screen	25	2a
Water_U^b^	Northwest England	Kox-U	ST236	Wastewater	−1	Remote site
Scot_T	Scotland	Kpn-T	ST147	Rectal screen	19	Remote site
Lon_S	London center 2	Eco-S	ST405	Rectal screen	+3	Remote site

Bacterial identifiers, including species codes, are shown alongside multilocus sequence types. Temporal cluster assignations were assigned around 2 clusters from the same hospital: 1, the historical cluster; 2, the cluster associated with the recognized index case; 2a, non–*Klebsiella oxytoca* isolated at the same National Health Service trust around the same time as cluster 2. The temporally diverse isolates from geographically remote sites in the United Kingdom are also shown (remote sites).

Abbreviations: BAL, bronchoalveolar lavage; Ecl, *Enterobacter cloacae*; Eco, *Escherichia coli*; ITU, intensive therapy unit, Kox, *Klebsiella oxytoca*; Kpn, *Klebsiella pneumoniae*; MLST, multilocus sequence typing; ST, sequence type.

**Figure 1.**
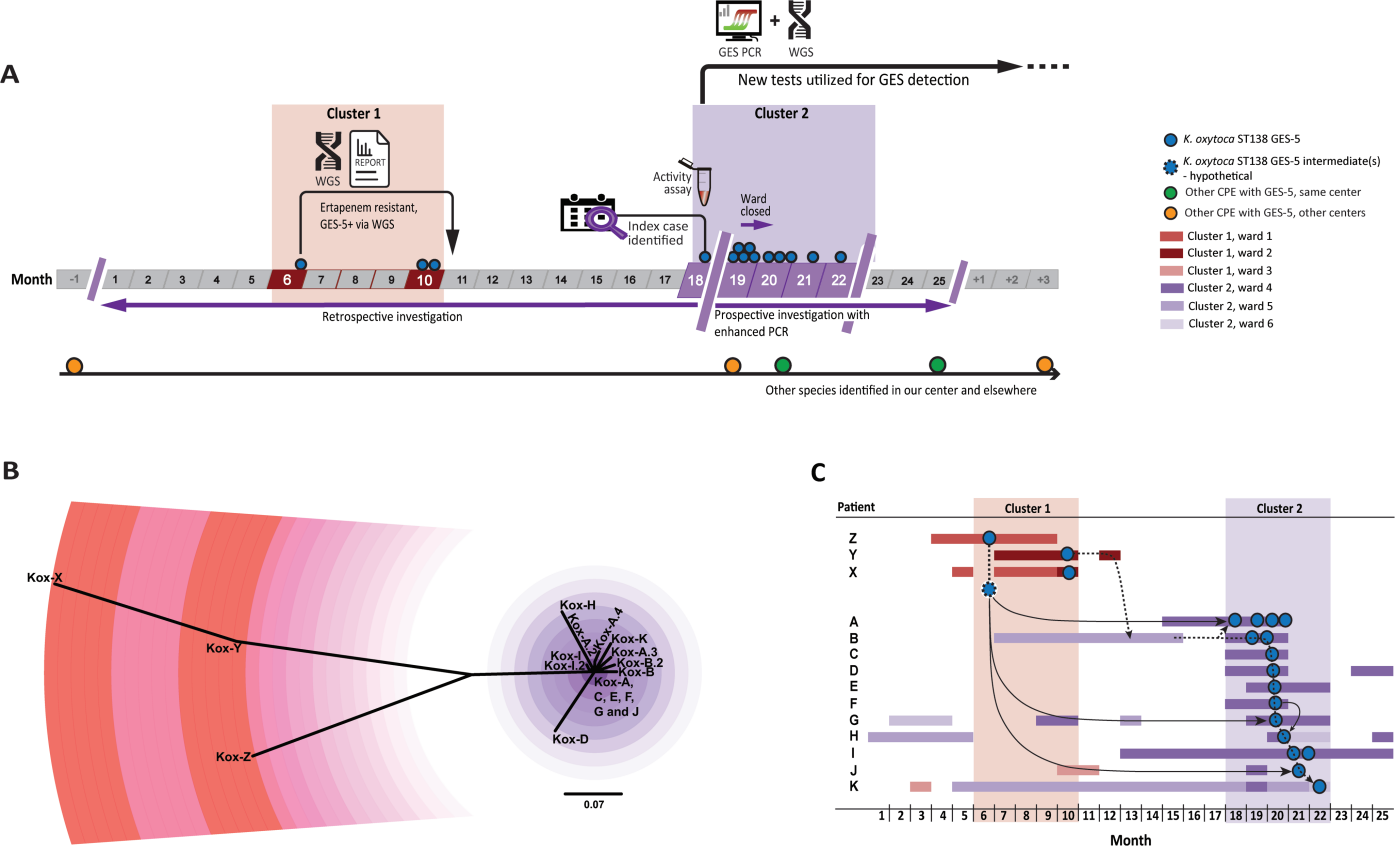
Two clusters of GES-5–producing *Klebsiella oxytoca* sequence type (ST) 138 occurred in patients at a single hospital over 18 months in 2 clinical departments. Events highlighted in red and purple shading indicate different clinical departments. The timeline (*A*) shows the temporal separation of the clusters along with major events for outbreak detection and management. The phylogenetic tree (*B*) based on WGS single-nucleotide polymorphism (SNP) analysis showed discrete genetic clustering of the isolates. Single SNPs are represented by graded coloration (purple or pink to distinguish between bacterial isolates from different clinical departments); the larger SNP distances in cluster 1 (red) are shown as alternating color blocks of 5 SNPs. The founding SNP profile for cluster 2 (purple) was shared by isolates *Klebsiella oxytoca* (Kox) A, G, and J. Transmission routes (*C*) derived from the accumulation of SNPs in patient isolates (in black arrows) refuted the hypothesized epidemiological links (dotted arrows) for a cascade of transmission in the second cluster, as well as proposed links between patient Y (dotted arrows) and the second cluster, but supported the isolate from patient Z as a progenitor for the founder of the second cluster. Abbreviations: CPE, carbapenemase-producing Enterobacterales; PCR, polymerase chain reaction; ST, sequence type; WGS, whole-genome sequencing.

^a^See timeline (Figure 1A).

^b^Published previously [19].

### Ethical Considerations

This work was classified as service evaluation and clinical investigation, and so was exempted from NHS Research Ethics Committee Review. The study of anonymized isolates beyond the diagnostic requirement was approved by an NHS research ethics committee (number 06/Q0406/20).

## RESULTS

### Detection of a GES-5 Producer Prompted Methodological Enhancements for the Detection of Unusual Carbapenemases

A urine isolate of *K. oxytoca* from patient Z (Kox-Z; see Table 1) was found to be ceftazidime and ertapenem resistant but negative for the major carbapenemase genes. WGS [28] identified a GES-5 carbapenemase gene, prompting the implementation of a GES PCR assay for rapid analysis of future phenotypically similar isolates of Enterobacterales (Figure 1A).

### Detection of a Rare Resistance Mechanism Triggered an Outbreak Investigation

The index case with a GES-5–producing *K. oxytoca*, which prompted the epidemiological investigation, was patient A located in a hematology unit (Table 1). The urine isolate Kox-A was detected using the GES-PCR; it occurred 1 year after Kox-Z in a different area of the hospital. Kox-A displayed ESBL activity and was resistant to ertapenem (MIC = 4 mg/L), but the major carbapenemase genes were not detected by PCR. Further to the detection of a GES-encoding gene, the isolate was found to have in vitro carbapenemase activity via Carba-NP (whereas Hodge testing of later isolates showed only equivocal activity; Supplementary Figure 3), and WGS confirmed the isolate encoded a GES-5 carbapenemase. Contact tracing and rectal screening of ward contacts was commenced. No recent travel history or contact with CPE carriers was identified. From the 102 ward contacts of patient A, 10 further GES-5–producing *K. oxytoca–*positive carriers were found (Table 1, patients B–K). No further cases of invasive *K. oxytoca* were identified in this cluster.

### A Missed Cluster Was Revealed

A look back exercise of the intervening period identified 6 additional ertapenem-intermediate or -resistant *K. oxytoca* isolates. Two isolates from distinct hospital sites were not referred and were no longer available, 2 were from the same hospital as the clusters but were not GES-5 producers, and the remaining 2 were PCR negative in the local laboratory but found to be GES-5 positive via WGS (Kox-Y and Kox-X, from patients Y and X, Table 1). Isolates from cases X, Y, and Z occurred over 18 weeks. Retrospective investigation revealed the 3 cases to be epidemiologically linked, and thus were designated as cluster 1. Cluster 1 occurred in the cardiothoracic and intensive therapy unit areas of the hospital, distinct and separate to the clinical area for cluster 2 (Table 1).

### Genomic Molecular Epidemiological Investigation

WGS confirmed that the 19 isolates (from the 11 patients in cluster 2 and the 3 patients in cluster 1; Figure 1A) were GES-5–producing *K. oxytoca* sequence type (ST) 138 (Table 1), with phenotypic antibiotic susceptibility profiles that could be explained by specific single-nucleotide polymorphisms (SNPs) and/or acquired antibiotic resistance genes (Supplementary Figure 1 and Supplementary Table 1). To determine the genetic relatedness between the isolates, the 19 ST138 *K. oxytoca* genomes were compared with a mapping-corrected assembly of the genome from the earliest isolate (Kox-Z, Table 1), which was chosen in the absence of a reference sequence for *K. oxytoca* ST138. Comparison of 6 306 956 bp of DNA from each isolate and phylogenetic analysis of the detected SNPs confirmed that the 19 isolates fell into 2 distinct clusters (Figure 1B and Supplementary Figure 1) that were separated by a minimum of 26 SNPs. This separation contrasted with the resolution and findings of the other epidemiological typing method, PFGE (Supplementary Figure 2). Further genomic analysis revealed greater diversity (ie, more SNPs occurring) among the 3 isolates within cluster 1 than between the 11 isolates in cluster 2, despite the similar duration of each cluster (5 months). For cluster 1, 43 SNPs separated the 3 isolates over 17 weeks, while the 11 isolates in cluster 2 were separated by 11 SNPs occurring over 20 weeks (Figure 1B). Epidemiological review revealed that patients B (cluster 2) and Y (cluster 1) were admitted to the hospital at the same time but did not share a clinical area for contact; the corresponding isolates Kox-Y and Kox-B were separated by 26 SNPs distributed across the genome (Supplementary Table 2). Further genomic analysis indicated that the isolate from patient Z harbored a progenitor of the SNP profile in the founder isolate for cluster 2 (Kox-A) as well as isolates from patients F, G, and I, which occurred over a 13-week period (Figure 1C). Where multiple sequential isolates were available from the same patient (patients A, B, and I in cluster 2), there was a maximum of 2 SNPs separating the initial and subsequent isolates from the same patient (Supplementary Table 2).

### A Multimodal Response Controlled Cluster 2

Following the identification of further cases to patient A (cluster 2) and the suggestion of local transmission, the ward was closed (Figure 1A) to admissions until no new isolations occurred from 3 consecutive patient rectal screens. After no new cases were identified, weekly rectal screening was continued in addition to admission rectal screening for all patients supported by molecular detection for GES when appropriate, practices which are still in place (at the time of writing).

### Multihospital Risk-based Screening Identified Additional Bacterial Genera Encoding GES-5

The 12-month period of prospective screening did not identify any further cases with *K. oxytoca* positive for GES-5, although 2 cases with GES-encoding *E. coli* (isolate Eco-V) and *E. cloacae* (isolate Ecl-W; Table 1) were detected. PCR and WGS confirmed the isolates were GES-5 gene carriers, but the patients were in separate clinical areas and had no known contact with any of the 14 cases from the *K. oxytoca* GES-5 clusters.

### Comparative Analysis and Observed National Spread of GES-5 Plasmid pHPRU111

The plasmid encoding the GES-5 gene in *K. oxytoca* ST138 (Kox-Z) (pJF707; KX946994) was identified as an 8300 bp IncQ plasmid, and was identical to the GES-5 plasmid confirmed by long-read (PacBio) sequencing of GES-5 positive *E. cloacae* (Ecl-W). The plasmid from Ecl-W was designated as pHPRU111 (MN180807) and used for comparison with other genomes of ST138 *K. oxytoca* and the *E. coli* ST66 within the study.

All GES-5 isolates from the hospital had identical plasmids, indicating a wider circulation of this plasmid among patients identified in the same hospital organization. We then set out to investigate if this plasmid was restricted to a single center or was more widely distributed across the UK. While other genetic vehicles for GES-5 were present among the GES-5 positive isolates in the reference collection of CPE, 3 isolates from the Northwest of England, Scotland, and a different London hospital had the pHPRU111 plasmid and occurred before (2014), during (2015), and after (2016) the clusters we detected (Table 1).

**Figure 2.**
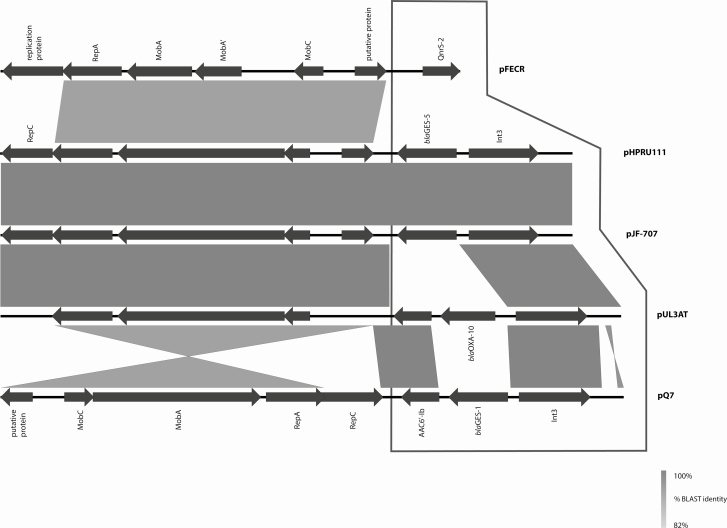
GES-5 encoding plasmids pHPRU111 (GES-5) and pJF-707 were unique in the nucleotide database (National Center for Biotechnology Information, GenBank, last accessed 27 December 2018). All comparators varied in the gene content of the integron and resistance regions (framed area). Backbone regions (nonframed area) varied in percentage identity from pUL3AT (*Enterobacter cloacae*, effluent, France 2007) and pFECR (uncultured bacterium, wastewater, Canada, circa 2017) and orientation relative to the resistance region of pQ7 (*Escherichia coli*, human, Switzerland, 1998). Abbreviation: BLAST, Basic Local Alignment Search Tool.

Other publicly available resistance-encoding plasmids of the same family (IncQ-based) as pHPRU111 were highly variable (Figure 2). In particular, the resistance gene content of pHPRU111 varied from each of pUL3AT (*E. cloacae*, effluent, France 2007), pQ7 (*E. coli*, human, Switzerland, 1998) and pFECR (uncultured bacterium, wastewater, Canada, circa 2017), for the presence or absence of *bla*_oxa-10_, *aac-(6′)-Ib,* GES-1 ESBL, and *qnrS* genes, respectively. On pQ7 the resistance region was integrated in the opposite orientation [29] (Figure 2).

## DISCUSSION

Carbapenemases threaten the effective treatment of gram-negative bacterial infections and create a diagnostic problem due to the diverse genotypes and sometimes cryptic phenotypes they present. Here, WGS unexpectedly identified a GES-5 CPE isolate from the original cluster (cluster 1). While WGS is a powerful tool in identifying novel or unexpected resistance mechanisms, current platforms rarely generate interpretable results within a clinically actionable timeframe. However, as we demonstrate, WGS can facilitate the development of rapid detection assays. The GES PCR enhanced the reference laboratory methods for detecting GES carbapenemases and led to rapid, real-time detection of an active cluster (cluster 2) of GES-5–producing *K. oxytoca*, as well as the retrospective identification of a missed cluster of GES-5 producers earlier in the same centre. The high discriminatory power of WGS gave insights into the progression of the clusters and their relationship to each other.

GES carbapenemases are relatively unusual globally [20], and in the UK [30], with only 34 isolates detected from 3541 CPE isolates confirmed by the national reference laboratory in 2014 and 2015 [11]. The initial delay in the detection of the GES-5 carbapenemase in this study was attributable to the relatively low carbapenem MICs of the isolates despite detectable phenotypic carbapenemase activity in vitro, as previously reported [19, 31], and the current lack of GES gene representation in most of the commercial molecular assays. These issues support the continued use of routine culturing and follow-up investigations to identify novel or unusual resistance mechanisms in an era of molecular diagnostics. Currently, multiplex PCR targeting 12 carbapenemase families, including GES, is carried out by the reference laboratory on all Enterobacterales submitted for investigation of carbapenem resistance [32], augmented by further phenotypic screening, including a carbapenemase activity assay applied to carbapenem-resistant isolates that are negative for the major carbapenemases, to help to detect novel and emerging carbapenemases. However the importance of using independent tests for detecting and confirming enzymes such as GES carbapenemases must be emphasized, given the variable reports for successful GES carbapenemase detection using phenotypic assays [19, 33]. While ceftazidime/avibactam was not tested at the time, targeted retesting has suggested that isolates encoding GES-5 (in the absence of a metallo-carbapenemase) were susceptible (Supplementary Table 2).

The multimodal response described controlled the outbreak during cluster 2; it utilized epidemiological investigations, enhanced infection control measures, improvements to the estate, and prospective screening supported by reference laboratory engagement with a carbapenemase activity assay and, crucially, an enhanced PCR for GES genes. Combined epidemiological, WGS, and laboratory data enabled us to conclude that 2 clusters of GES-5 producers were driven by the same genotype (ST138) of *K. oxytoca* carrying an identical GES-5 encoding plasmid. Together with other observations of resistant ST138 *K. oxytoca* [34], this work highlights the lineage as a “high-risk,” internationally disseminated, persistent clone that poses a threat to hospitalized patients. The work is also the first report of outbreaks and infections due to GES-5 carbapenemase-encoding *K. oxytoca* and is consistent with increasing concerns over *K. oxytoca* as an emerging carbapenemase-producing pathogen [34].

The availability of WGS at the national reference center also provided unexpected molecular epidemiological information that was not apparent from the PFGE data. The greater SNP diversity observed between isolates in cluster 1 may result from the presence of an unsampled but more diverse reservoir of *K. oxytoca* isolate variants from which the patients acquired their disease-causing isolates. No single patient could be identified as the epidemiological bridge between the 2 *K. oxytoca* ST138 clusters; an epidemiological cascade of transmission in cluster 2 and the SNP diversity within the cluster suggested that an unidentified common source(s) harbored the variants of ST138 that colonized the patients in this cluster. This (unidentified) source could have been an environmental reservoir, an unidentified carrier, or ongoing (and undetected) transmission between patients due to a breakdown of infection control practices. Noted associations between *K. oxytoca* and sinks, water, and wastewater systems [19, 21, 35] did prompt nonexhaustive environmental investigations and such sites remain a consideration for subsequent investigations. A better understanding of the interactions between patients, staff, and the healthcare environment, along with enhanced sampling strategies for patients and their environment, would improve the resolution for reconstructing and understanding transmission routes.

Our contextual, national-level analysis, using short-read (Illumina) and targeted long-read (PacBio) sequencing, showed the unusual nature of the carbapenem resistance (GES-5 gene) and its (IncQ group) plasmid vector and revealed the wide distribution across other species and genera within the hospital group and across the UK. It also enabled us to determine that this plasmid is not the only genetic vector of GES-5 gene present in the UK. The relationship of pHPRU111 to its best match in the public database, the GES-1 ESBL-encoding pQ7 found at least 5 years earlier in Switzerland [29], reflects the persistence, success, and wider circulation of this plasmid backbone and the need to monitor its further spread.

Overall, our findings highlight the importance of vigilance for unusual carbapenemases, especially those that can be difficult to detect, and underlines the risk of covert plasmid propagation in healthcare settings. The use of WGS for characterizing CPE provided clear benefits for the control of this outbreak, demonstrating the translational potential of this technology for routine use. The case for better resourcing of the detection and characterization of CPE to minimize their spread lies not only in the interests of patients but also healthcare providers who bear the significant costs associated with CPE outbreaks [13].

## Supplementary Data

Supplementary materials are available at *Clinical Infectious Diseases* online. Consisting of data provided by the authors to benefit the reader, the posted materials are not copyedited and are the sole responsibility of the authors, so questions or comments should be addressed to the corresponding author.

ciz1130_suppl_Supplementary_Materials

ciz1130_suppl_Supplementary_Tables

## Notes

***Author contributions.* **M. J. E., F. D., E. J., K. L. H., J. A. O., S. S., N. W., and A. H. conceived the study. F. D., G. A., J. P., A. J. I., C. E., E. B., T. K., Y. C., B. P., S. M., J. A. O., S. S., and A. H. oversaw hospital epidemiology. F. D., G. A., S. S., S. M., and J. A. O. oversaw clinical microbiology. K. L. H. and J. F. T. performed molecular analyses such as pulsed-field gel electrophoresis. R. H. oversaw antimicrobial susceptibility testing. M. J. E., F. D., and E. J. performed data analysis and table preparation. M. J. E. carried out genome sequencing analysis and table and figure preparation. J. F. and L. W. contributed genomic sequences of isolates from other centers. M. J. E., F. D., and E. J. wrote the manuscript. All coauthors contributed to further versions of the manuscript.

***Acknowledgments.* **The authors thank Nazim Mustafa for the preparation of DNA for Illumina sequencing at Public Health England (PHE), and PHE’s Genomic Services and Development Unit for the provision of short-read sequence data; Professor Derrick Crook and the University of Oxford National Institute for Health Research (NIHR) Health Protection Research Unit (HPRU) on Healthcare Associated Infection and Antimicrobial Resistance for assisting with the preparation of DNA for long-read PacBio sequencing by Dr Robert Sebra and the team at the Mount Sinai Institute (New York); and Dr Hugo Donaldson at North West London Pathology for Hodge test images.

***Disclaimer.* **The funders had no role in the study design; in the collection, analysis, and interpretation of data; in the writing of the report; or in the decision to submit the paper for publication. The views expressed are those of the authors and not necessarily those of the National Health Service, the NIHR, the Department of Health, or PHE.

***Financial support.* **This work was funded by the NIHR HPRU in Healthcare Associated Infections and Antimicrobial Resistance at Imperial College London in partnership with PHE, in collaboration with the Sanger Institute, the University of Cambridge Veterinary School, and Imperial College Health Partners. S. S. and A. H. also acknowledge the support of the NIHR Imperial Biomedical Research Centre. A. H. is an NIHR Senior Research Investigator. E. J. is an Imperial College Research Fellow jointly funded by Rosetrees Trust and the Stoneygate Trust (M683).

***Potential conflicts of interest.*** M. J. E., K. L. H., R. H., J. F. T., and N. W. are members of PHE’s Antimicrobial Resistance and Healthcare Associated Infections Reference Unit, which has received financial support for conference attendance, lectures, research projects, or contracted evaluations from numerous sources, including Accelerate Diagnostics, Achaogen Inc, Allecra Therapeutics, Amplex, AstraZeneca UK Ltd, AusDiagnostics, Basilea Pharmaceutica, Becton Dickinson Diagnostics, bioMérieux, Bio-Rad Laboratories, British Society for Antimicrobial Chemotherapy, Cepheid, Check-Points B.V., Cubist Pharmaceuticals, Department of Health, Enigma Diagnostics, the European Centre for Disease Prevention and Control, Food Standards Agency, GenePOC, GlaxoSmithKline Services Ltd, Helperby Therapeutics, Henry Stewart Talks, International Health Management Associates Ltd, Innovate UK, Kalidex Pharmaceuticals, Melinta Therapeutics, Merck Sharpe & Dohme, Meiji Seika Pharma Co Ltd, Mobidiag, Momentum Biosciences Ltd, Neem Biotech, NIHR, Nordic Pharma Ltd, Norgine Pharmaceuticals, Paratek, Rabiotics Rx, Rempex Pharmaceuticals Ltd, Roche, Rokitan Ltd, Smith & Nephew UK Ltd, Shionogi & Co Ltd, Tetraphase Pharmaceuticals, Trius Therapeutics, VenatoRx Pharmaceuticals, Wockhardt Ltd, and the World Health Organization. J. A. O. has consulted for Gama Healthcare Ltd and Pfizer Ltd in the past 3 years, outside the submitted work. All other authors report no potential conflicts of interest. All authors have submitted the ICMJE Form for Disclosure of Potential Conflicts of Interest. Conflicts that the editors consider relevant to the content of the manuscript have been disclosed.
