# Supplementary material for: A Multispecies Cluster of GES-5 Carbapenemase–Producing Enterobacterales Linked by a Geographically Disseminated Plasmid
Source: Clin Infect Dis. 2019 Nov 20;71(10):2553–60. doi: 10.1093/cid/ciz1130 (PMC7744980; doi:10.1093/cid/ciz1130)

Supplementary Materials

**Supplementary Materials and Methods**

*Patient screening and laboratory detection of GES producers*

At the time the detected outbreak occurred, universal admission and weekly screening was in place in high risk areas (e.g. the ICU) and risk factor-based screening of patients, in other areas of the hospital. The risk factors were those described by Public Health England: Individuals who, in the last 12 months, were (a) an inpatient in a hospital abroad or (b) an inpatient in a UK hospital with known spread of carbapenemase producing Enterobacteriaceae or (c) were ‘previously’ positive for CPE [1].

In the clinical diagnostic laboratory, rectal or faecal screening isolates were plated onto Colorex^TM^ mSuperCARBA^TM^ screening agar (E&O Laboratories) and clinical isolates were processed according to local standard operating procedures, including antimicrobial disc susceptibility testing and species identification via MALDI-TOF MS (Bruker Daltonics). Susceptibilities were interpreted according to EUCAST criteria with ertapenem results of intermediate or resistant according to EUCAST criteria [2] treated as an indicator of putative carbapenemase production. Where molecular diagnostic PCR detection was negative for OXA-48-like, NDM, VIM, IMP and KPC carbapenemase genes (Cepheid Xpert® Carba-R) isolates were treated as suspicious for production of an unusual carbapenemase and referred to the national reference laboratory. An enhanced protocol that included a PCR which targeted GES genes [3] was used for all such suspicious Enterobacterales from this centre, and a carbapenemase activity (RAPIDEC CarbaNP, Biomerieux, France) was implemented including Kox-Z, by the reference laboratory. These investigations were carried out in addition to reference MIC determinations via agar dilution for an extended panel of antimicrobials and supplemented the outbreak investigation.

*Genomic analysis*

For WGS DNA was prepared using the QiaSymphony DNA extraction platform (Qiagen). The Nextera XT DNA Library Preparation kit (Illumina, USA) was used to prepare multiplexed DNA sequencing libraries. Whole genome sequencing was undertaken using the Illumina HiSeq 2500 System (Illumina, US) and 2 x 100bp paired-end mode. PacBio sequencing of QiaSymphony (Qiagen) extracted DNA was performed on a PacBio RSII using P6/C4 sequencing chemistry. Libraries were made using the SMRTbell Template Prep Kit 1.0. Sequence data have been deposited in the European Nucleotide Archive (Table S1). An identical plasmid sequence was assembled (using SPAdes) [4] after the GES-5 plasmid was transferred from Kox-A to *E. coli* DH5α.

A reference genome internal to the outbreak, chosen as the first available isolate (Kox-Z, from patient Z) was also assembled using SPAdes. Contigs smaller than 500bp were removed from the assembly. The plasmid contig was added to the assembly. Sequence reads from Kox-Z were mapped against the assembly using the PHEnix workflow (<http://phenix.readthedocs.io/en/latest/>). Briefly, BWA was used as the read mapping software, (version 0.7.9a), the SAM files generated were converted to BAM with Samtools (version 1.1) and single nucleotide polymorphisms (SNPs) called using the Genome Analysis Toolkit 2 (GATK2), with filtering based on the depth of coverage (DP ≥ 5), ratio of unfiltered reads that support the reported allele compared to the reference (AD ≥ 0.8) and mapping quality (MQ ≥ 30). The filtered SNPs compared to assembly, including heterozygotes, which were designated as ‘N’, were combined to generate a single multiple alignment file with the maximum proportion of Ns accepted at any position of the alignment set to less than 20%. The SNPs detected in the initial assembly of Kox-Z were corrected according to the corresponding VCF file. The resulting corrected reference was used for comparisons against the genomic reads from each isolate using the PHEnix workflow, as above. The maximum likelihood (ML) tree was constructed using RaxML [5] MLST was determined using MOST [6], resistance genes, plasmid replicon types and the presence of the 8300bp pHPRU111 plasmid was also determined using PHEgenefinder [7, 8]. The presence of the plasmid pHPRU111 was confirmed in Mauve, and VCFs checked for SNPs in positions that corresponded to plasmid sequence. Plasmids identified as related to pHPRU111 (>80% nucleotide similarity with a 80% match across the length of pHPRU111) were downloaded (pQ7 FJ696404; pUL3AT HE616889 and pFECR MF554639) and compared with pJF-707 and pHPRU111 using BLASTn and visualised using EasyFig [9].

**References**

1. Public Health England. Acute trust toolkit for the early detection, management and control of carbapenemase-producing Enterobacteriaceae. UK Government DoH. **2013**; <https://www.gov.uk/government/publications/carbapenemase-producing-enterobacteriaceae-early-detection-management-andcontrol-toolkit-for-acute-trusts> Last accessed: 01/08/2019.

2. The European Committee on Antimicrobial Susceptibility Testing. Breakpoint tables for interpretation of MICs and zone diameters. Version 5.0, 2016. **2016**; <http://www.eucast.org/fileadmin/src/media/PDFs/EUCAST_files/Breakpoint_tables/v_5.0_Breakpoint_Table_01.xls>; Last accessed: 24/03/2019.

3. Poirel L, Le Thomas I, Naas T, Karim A, Nordmann P. Biochemical sequence analyses of GES-1, a novel class A extended-spectrum beta-lactamase, and the class 1 integron In52 from Klebsiella pneumoniae. Antimicrob Agents Chemother **2000**; 44(3): 622-32.

4. Bankevich A, Nurk S, Antipov D, et al. SPAdes: a new genome assembly algorithm and its applications to single-cell sequencing. J Comput Biol **2012**; 19(5): 455-77.

5. Stamatakis A. RAxML version 8: a tool for phylogenetic analysis and post-analysis of large phylogenies. Bioinformatics **2014**; 30(9): 1312-3.

6. Tewolde R, Dallman T, Schaefer U, et al. MOST: a modified MLST typing tool based on short read sequencing. PeerJ **2016**; 4: e2308.

7. Doumith M, Godbole G, Ashton P, et al. Detection of the plasmid-mediated mcr-1 gene conferring colistin resistance in human and food isolates of Salmonella enterica and Escherichia coli in England and Wales. J Antimicrob Chemother **2016**; 71(8): 2300-5.

8. Neuert S, Nair S, Day MR, et al. Prediction of Phenotypic Antimicrobial Resistance Profiles From Whole Genome Sequences of Non-typhoidal Salmonella enterica. Front Microbiol **2018**; 9: 592.

9. Sullivan MJ, Petty NK, Beatson SA. Easyfig: a genome comparison visualizer. Bioinformatics **2011**; 27(7): 1009-10.

**Supplementary Tables and Figures**

**Supplementary table S1:** FASTQ accessions (ENA BioProject PRJEB30858) and microbiological features, including the required minimum inhibitory concentrations of antimicrobials and the resistance alleles detected.

**Supplementary table S2.** SNP profiles, positions of SNPs and annotated coding sequences of isolates. Relative to a *de novo* assembly of the initial ST138 *K. oxytoca*, Kox-Z.

**Figure S1. Cladogram related plot of predicted resistances based on genotypic detection of resistance genes and mutations, compared with antimicrobial susceptibility phenotype categorisations.** Antimicrobials are shown all in upper case. Genes/loci encoding resistance are shown (red), blank spaces (grey) in columns indicate no detection of a resistance locus. Phenotypic categorisations are given according to EUCAST 2016 breakpoints [2] as ‘S’usceptible (green), ‘I’ntermediate (yellow) or ‘R’esistant (red), synergies with beta-lactamase inhibitors (clavulanate or EDTA) are shown as positive (+) or negative (-).


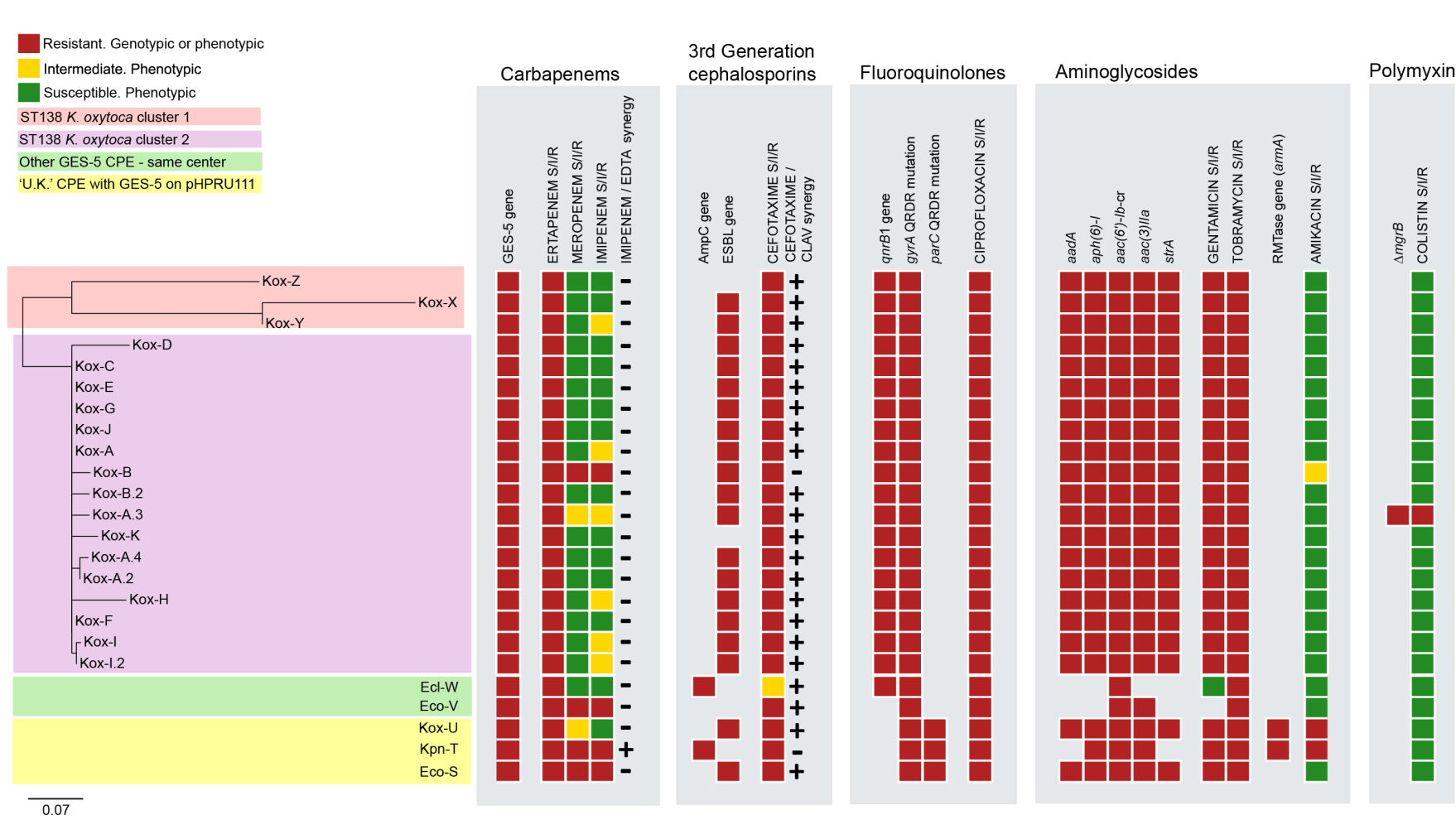


**Figure S2. PFGE profiles of *Xba*I-digested *K. oxytoca* isolated from the 14 patients A-K and X-Z in different clinical areas (purple vs. red overlay indicating Clusters 2 and 1, respectively) within a single centre indicated the clonality of the cluster isolates.**


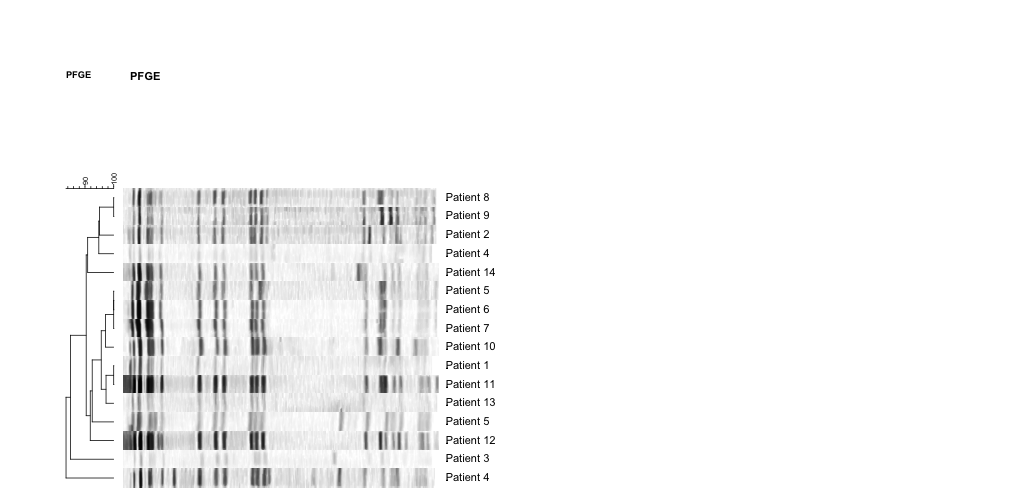


% identity

(band profile)

Kox-E

Kox-F

Kox-Y

Kox-B

Kox-K

Kox-A

Kox-C

Kox-D

Kox-G

Kox-Z

Kox-H

Kox-J

Kox-A.3

Kox-I

Kox-X

Kox-B.2

­

**Figure S3. Modified Hodge test of isolate Kox_A (indicated with arrows), showing weak or very weak positivity as deformation of the zone of inhibition around 10 µg ertapenem (ETP) or impenem (IPM) discs.** The negative control *Klebsiella pneumoniae* ATCC700603 and positive control *Klebsiella pneumoniae* OXA48 NCTC13442 showed no deformation or strong deformation of the zone of inhibition, respectively of background *E. coli* ATCC25922. No positivity was seen using meropenem (not shown).


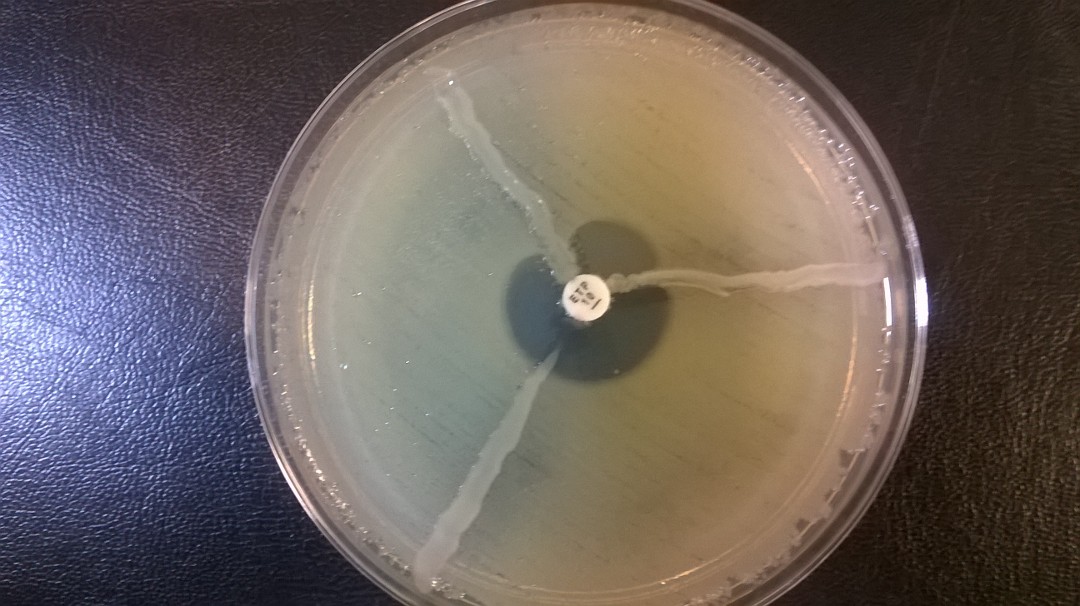

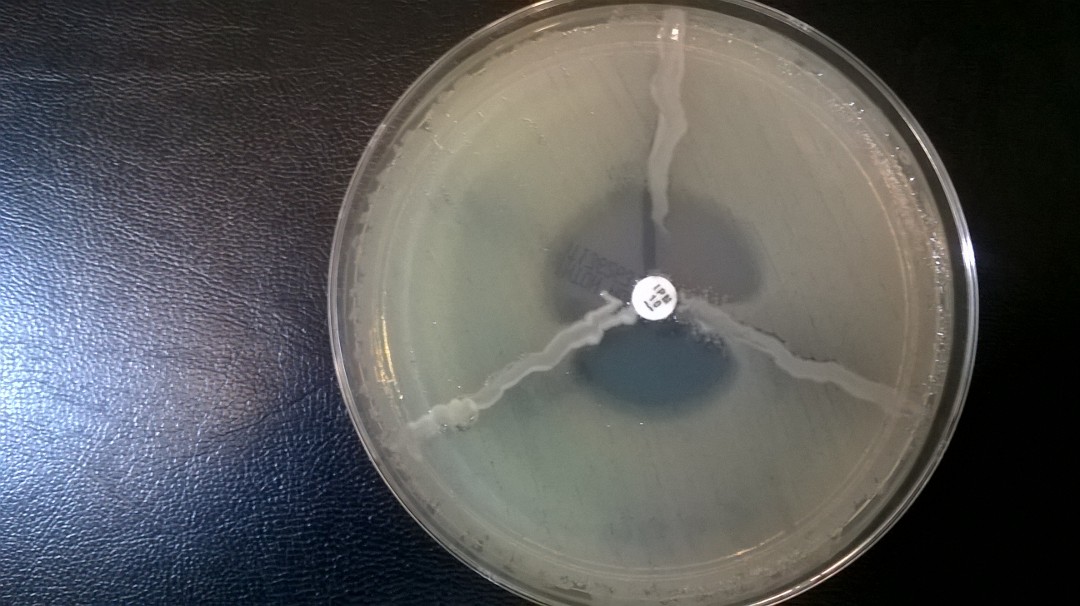

Supplement: ciz1130_suppl_Supplementary_Materials [file ciz1130_suppl_supplementary_materials.docx]
